# Translating an Afro-European partnership into tangible hand hygiene action

**DOI:** 10.1186/1753-6561-5-S6-P263

**Published:** 2011-06-29

**Authors:** S Bagheri Nejad, M-N Chraiti, J Carlet, J Hightower, SB Syed

**Affiliations:** 1 World Health Organization, Geneva, Switzerland

## Introduction / objectives

WHO African Partnerships for Patient Safety (APPS) nurtures sustainable partnerships between African and European hospitals to improve patient safety. Through APPS, Hôpitaux Universitaires de Genève (HUG) is partnered with hospitals in Cameroon, Mali and Senegal.

## Methods

Hospital representatives convened in 2009 to build the programme foundation, followed by hospital situational analyses to determine priority action areas. Among 12 patient safety action areas, reduction of health care-associated infection (HAI) was chosen as a common platform of action. Subsequently, 2-year partnership action plans were developed, finalized at a workshop in 2009 in Uganda. Hand hygiene (HH), the single most effective measure to reduce HAI, was the cornerstone of planned action.

## Results

A broad range of HH improvement tools was made available through a patient safety resource map. African technicians were trained on alcohol-based handrub production. An initial batch of materials was provided for African hospitals to enable local production. A HH training workshop was held in September 2010 at HUG led by hospital infection control experts. African participants were designated HH project coordinator, trainer and observer. In-depth training on the WHO multimodal hand hygiene improvement strategy and WHO's hand hygiene observation method took place, accompanied by practical sessions on HH technique, displaying training films, and educational games. The workshop also provided an opportunity for collective brainstorming, exchange of knowledge and common issues, and sharing on-the-ground experience across two continents.

## Conclusion

APPS has created a mechanism to translate inter-continental partnerships into tangible action on improving hand hygiene in order to reduce HAI.

## Disclosure of interest

None declared.

